# Fertility trends by HIV status in a health and demographic surveillance study in Magu District, Tanzania, 1994–2018

**DOI:** 10.1371/journal.pone.0281914

**Published:** 2023-02-21

**Authors:** Denna Mkwashapi, Jenny Renju, Michael Mahande, John Changalucha, Mark Urassa, Jim Todd

**Affiliations:** 1 Sexual and Reproductive Health Department, National Institute for Medical Research, Mwanza, Mwanza, United Republic of Tanzania; 2 Department of Epidemiology and Biostatistics, Kilimanjaro Christian Medical University College, Moshi, Tanzania, The United Republic of Tanzania; 3 Department of Population Health, London School of Hygiene and Tropical Medicine, London, England; University of Washington, UNITED STATES

## Abstract

**Background:**

Sub-Saharan Africa (SSA) has the highest fertility rates and highest HIV disease burden globally. However, it is not clear how the rapid expansion of anti-retroviral therapy (ART) for HIV has impacted the fertility gap between HIV-infected and uninfected women. We used data from a Health and Demographic Surveillance System (HDSS) in north-western Tanzania to explore trends in fertility rates and the relationship between HIV and fertility over the 25-year period.

**Methods:**

From 1994 to 2018, births and population denominators from the HDSS population were used to obtain age-specific fertility rates (ASFRs) and total fertility rates (TFRs). HIV status was extracted from eight rounds of epidemiologic serological surveillances (1994–2017). Fertility rates by HIV status and in different levels of ART availability were compared over time. Independent risk factors for fertility changes were examined using Cox proportional hazard models.

**Results:**

There were 24,662 births from 36,814 women (15–49) who contributed a total of 145,452.5 Person-Years of follow-ups. The TFR declined from 6.5 births per woman in 1994–1998 to 4.3 births per woman in 2014–2018. The number of births per woman was 40% lower in women living with HIV than in HIV-uninfected women (4.4 vs. 6.7), although this difference narrowed over time. The fertility rate in HIV-uninfected women was 36% lower in 2013–2018 than in 1994–1998(age-adjusted HR = 0.641; 95% CI 0.613–0.673). In contrast, the fertility rate in women living with HIV was relatively unchanged over the same follow up period (age-adjusted HR = 1.099; 95% CI 0.870–1.387).

**Conclusions:**

There was a notable fertility decline among women in the study area from 1994 to 2018. Fertility remained lower in women living with HIV than in HIV-uninfected women, but the difference continued to narrow over time. These results highlight the need for more research into fertility changes, fertility desire and family planning use in Tanzanian rural communities.

## Introduction

The United Nations estimated a significant decline in fertility across the globe from 2010–2019. Sub-Saharan Africa (SSA) has the highest fertility rates in the world, at 5.1 births per woman, while total fertility rates in the rest of world was 2.5 births per woman in 2010–2019 [1]. In 2015, Tanzania was ranked as having the second-highest fertility in East Africa, with 5.2 births per woman [2]. General fertility rates in north-western Tanzania between 1994 and 1998 were reported to be 203 births per 1000 woman-years, and higher fertility was reported among women in rural areas, those with lower socioeconomic status and lower educational levels [3]. High fertility contributes to a greater risk of maternal and child mortality during childbearing [4].

Countries in SSA are experiencing not only higher fertility but also a higher HIV disease burden than the rest of the world. The region has the highest HIV prevalence and incidence in the world [5]. In 2017, the national prevalence of HIV/AIDS in Tanzania was 4.6%, with an incidence of 1.4 new infections per 1,000 adults aged 15–49 years [5, 6]. HIV infection affects fertility through biological, behavioural, and social mechanisms. Biologically, fertility is lowered in women living with HIV due to an increased risk of spontaneous abortion and stillbirths, increased amenorrhea and decreased coital frequencies in advanced HIV disease [7]. Ulcerative sexually transmitted infections (STIs), which are common in adults with advanced HIV disease, lower the likelihood of conception and pregnancy [8]. There are also social-behavioral reasons for lower fertility in women living with HIV, which include higher rates of widowhood and divorce with lower remarriage rates and increased use of condoms in both extramarital and marital unions [9–12].

Over the past two decades, the relationship between HIV infection and fertility has been widely studied, with lower fertility rates being found in women living with HIV [13]. Studies in Uganda, the Democratic Republic of Congo, and Cote d’Ivoire reported reductions in the general fertility rate of 15–47% among women living with HIV compared to HIV-uninfected women [14–16]. Following the availability of antiretroviral treatment (ART) in SSA, evidence suggests a fertility rebound amongst HIV-infected women. In a systematic review of studies looking at the impacts of ART on fertility in SSA, it was reported that ART use and duration on ART were associated with increased fertility rates [17, 18]. However, conflicting results have been reported. Some studies have reported a lower pregnancy incidence among HIV-infected women on ART than among those not on ART [19, 20], while others have reported no statistically significant difference between these groups [21].

Since 2004, Tanzania started to provide ART to eligible HIV-infected patients. In 2013, lifelong ART was provided to all pregnant women diagnosed with HIV regardless of their disease stage and viral or CD4 cell count. This was called Prevention of Mother to Child Transmission of HIV—Option B+ [22]. The universal HIV test and treat (UTT) policy was later adopted in Tanzania (2016) and provided ART to all HIV-infected individuals regardless of their immune status [23].

The population impact of HIV and/or ART on fertility is more pronounced in countries with high HIV prevalence, HIV testing rate, and ART coverage [12]. In Tanzania, the HIV testing rate and ART coverage are both 82% among women aged 15 years and above in 2015/16 [24]. It is unclear how fertility by HIV infection has evolved alongside the different phases of the HIV epidemic and how overall fertility levels and trends have been affected by expanded access to ART.

In this analysis, we used data from the Magu Health and Demographic Surveillance System (HDSS) in Tanzania to explore trends in fertility rates over 25 years of follow-up from 1994 to 2018 and to determine the longer-term impact of HIV on women’s fertility rates during pre- and post-ART-expanded program implementation.

## Method

### Study setting

The study was carried out within the Magu Health and Demographic Surveillance System (HDSS), Tanzania. Magu HDSS started in 1994 to date and is one of oldest community-based cohorts in Sub-Saharan Africa [25]. By 2020, nine villages with a combined resident population of 45,000 people were included in the Magu HDSS. The study area lies 20 km east of Mwanza City, the region’s capital. The population is predominant rural with a single peri-urban trading centre. By 2020, HIV treatment services including PMTCT and family planning services have been provided at the wards’ referral health centre and four village dispensaries.

### Data collection

Information on the HDSS, serological surveillance systems and HIV testing procedures have been described in detail elsewhere [26, 27], but briefly, the demographic data for this study were drawn from 35 rounds of household visits, nearly 0.7 years apart from 1994 to 2018, capturing all births and deaths in the resident population. HIV status data for this study were also drawn from eight rounds of HIV epidemiologic and serologic surveillance, which was conducted every three years, from 1994 to 2017. Among all HDSS population, resident adults aged 15 years and above were invited to participate in the HIV epidemiologic and serologic surveillance. After informed consent was obtained, participants provided blood samples for anonymous HIV research testing and underwent a detailed face-to-face interview covering sexual behaviour, child-bearing, and the use of family planning and HIV services. Blood samples were tested for HIV at the National Institute for Medical Research (NIMR) reference laboratory in Mwanza.

### Data management

Residency episodes were defined for each period spent in the Magu HDSS and used to calculate the person-years (PY) for resident women in this analysis. The Magu HDSS defines residency as living for three months or more in the study area. All residency episodes started at birth, or date first seen in Magu HDSS, and finished at death, the date was last seen or the right censoring date (December 31, 2018) were captured. Data on the mother-to-child linkage, which linked every child and their birth dates to their mother, using the residency episodes to determine eligibility for the analysis.

Education was analysed as *a time-fixed variable* using the highest level of education attended for each woman, categorized into four levels: no education, primary 1–4 years, primary 5–7 years, and post primary education. Standard five-year age groups were used to present age-specific fertility rates (ASFRs), and women moved from one age group to the next as they aged. Calendar years were also grouped into five-year periods.

The HIV status of each woman was obtained from the HIV test results in the serological surveillance systems. The date of HIV seroconversion was estimated to be the midpoint between the first positive and last negative HIV test. An unknown HIV status was defined for women who had never been tested within the study area; if a woman had no previous negative tests, then the time one year before their first positive test was included as HIV positive, and the time before that classified as unknown HIV status. For women who were HIV-negative, we classified them as negative for five years after their last negative test and after that as HIV unknown.

The area of residence was classified as ’rural’ for remote villages and sub villages located within rural communities and ’peri-urban’ for the villages and sub villages residing around the trade centre. Dates of in- and out-migration were recorded for women who moved household and changed residence. Data entry and management were performed by using the Census and Survey Processing System (CSPro software) version 6.3.

### Statistical consideration

The age-specific fertility rate (ASFR) was defined as the number of live births to women divided by the number of person-years contributed by women aged 15–49 years, usually expressed in 5-year age intervals. Total fertility rates (TFRs) were defined as the sum of the ASFRs for women aged 15–49, interpreted as the average number of live births of the woman in her reproductive years (15–49).

From the ASFR calculation, we calculated TFR by calendar years, rural and peri-urban residence, educational level, and HIV status. In assessing the impact of ART on population fertility, we compared the TFR of women aged 15–49 over 5 years before ART availability (1994–1998); within 5 years before ART availability (1999–2003); during the introduction of ART (2004–2008): during ART availability (2009–2013) and finally during the period of provision of PMTCT- Option B+ (2013–2018) within the HDSS.

In the statistical methods, we used Poisson regression, to estimate fertility rates and rate ratios, and multivariable Cox proportional hazard models to estimate Adjusted hazard ratios (HRs) and 95% confident intervals (CI). In this model, we investigated the interaction between HIV status and five-year period. Wald test was used to assess significance of interaction terms. The final models included the effect of place of residence, time periods, HIV status. educational level, ART periods with and without the interactions. Analysis was performed using Stata, version 13.0 (Stata Corp, College Station, TX) statistical package.

### Ethical consideration

Ethical approval was obtained from the Lake Zone Institutional Review Board (MR/53/100/513) and the Ethical Review Committee of Kilimanjaro Christian Medical College of the Tumaini University of Tanzania (certificate number 2440).

## Results

Fertility data from 36,814 women aged 15–49 years between January 1994 and December 2018 were included for analysis, and a total of 145452.5 person-years (PYs) were observed. Women living with HIV contributed 4.6% of the total follow-up time, HIV negative women 60.4% of the time, while women of an unknown HIV status contributed 35% of the total follow-up time. The study observed a total of 24 662 births, of which 811 births were from women living with HIV (Table 1).

**Table 1:** Total fertility rates (TFR) among women aged 15–49 from January 1994 to December 2018.

Variable	Category	Births	Person years (PYs)	Total fertility rate (TFR)	General fertility Rate ratio^2^	95% Confidence Intervals (95% CI)	LRT p- value^3^
**All**	1994–2018	24662	145452.5	5.5	-	-	-
**Calendar year** ^**1**^							
	1994–1998	3901	18745.3	6.51	Ref		
	1999–2003	4889	23773.4	6.49	0.985	0.944–1.027	0.476
	2004–2008	5148	28054.3	5.99	0.880	0.844–0.918	<0.001
	2009–2013	5321	31727.1	5.59	0.807	0.774–0.841	<0.001
	2014–2018	5403	43154.8	4.25	0.580	0.555–0.607	<0.001
**Age groups**							
	15–19	3531	33446.2	0.53	Ref		
	20–24	6284	26157.2	1.20	2.286	2.190–2.386	<0.001
	25–29	5688	22712.8	1.25	2.387	2.285–2.494	<0.001
	30–34	4594	20482.4	1.12	2.126	2.032–2.226	<0.001
	35–39	3058	17301.1	0.89	1.685	1.603–1.772	<0.001
	40–44	1238	14146.0	0.44	0.835	0.781–0.893	<0.001
	45–49	269	11209.3	0.12	0.227	0.201–0.257	<0.001
**Place of Residence**							
	Rural	16191	79469.2	6.66	Ref		
	Peri urban	8471	65985.7	4.20	0.589	0.570–0.608	<0.001
**Educational level**							
	None	4387	23574.9	6.85	Ref		
	Primary 1–4	2238	10479.8	7.33	1.136	1.078–1.197	<0.001
	Primary 5–7	11109	53368.0	6.48	1.112	1.073–01.154	<0.001
	Secondary and Tertiary	1008	10867.2	3.49	0.498	0.448–0.194	<0.001
**HIV status**							
	HIV-Negative	16295	82277.2	6.72	Ref		
	HIV-Positive	811	6535.5	4.41	0.617	0.574–0.663	<0.001
	Unknown HIV Status	7137	56642.3	4.03	0.609	0.591–0.628	<0.001
**ART periods** ^**4**^							
	>5 years Pre ART	4840	23016.3	6.69	Ref		
	5–0 years Pre ART	5201	26172.6	6.30	0.945	0.910–0.982	0.004
	ART availability	8276	46455.8	5.94	0.863	0.831–0.896	<0.001
	ART & Option B+	6345	49810.2	4.33	0.612	0.589–0.635	<0.001

^1^Calender year is grouped into five groups of five years each

^2^Fertility rate ratios are " crude " and derived from univariate Poisson regression models

^3^Likelihood ratio test p-value

^4^ART periods are calendar years grouped into four groups;>5 years Pre-ART (1994–2000), 5–0 years Pre-ART (2001–2005), ART availability (2006–2012): ART & Option B+ (2013–2018)

### Total fertility rates and trends over time

The overall TFR for the entire 25 years of follow-up was 5.5 births per woman. There was a consistent decline in the TFR over the calendar years, from 6.51 births per woman in 1994–1998 to 4.25 births per woman in 2014–2018. This is equivalent to a 42% reduction in TFR from 1994 to 2018 (HR = 0.580; 95% CI 0.555–0.607). TFRs trends by calendar years, age groups, educational levels and place of residence are also shown (Table 1).

Over the 25 years of follow-up, women in rural areas had a TFR of 6.7 births per woman, while women dwelling in peri-urban areas had a TFR of 4.2 births per woman, representing a 38% difference in TFR (HR = 0.62; 95% CI: 0.61–0.65; p<0.001). In 1994–1998, with 40% women residing in peri-urban areas, there were 2.2 births per woman differences in the TFR between peri-urban and rural women, while in 2014–2018, with 47% women residing in peri-urban areas, the difference in the TFR between peri-urban and rural women was 2.0 births per woman. Similarly, the proportion of women with secondary education rose from 4% in 1994–1998 to 16% in 2014–2018, with a difference in TFR of 2.1 births per woman between women with secondary education and those with lower education. In 1994–1998, the difference in TFR between women with secondary education and those with lower education was 3.3 births per woman (Table 1).

**Fig 1:**
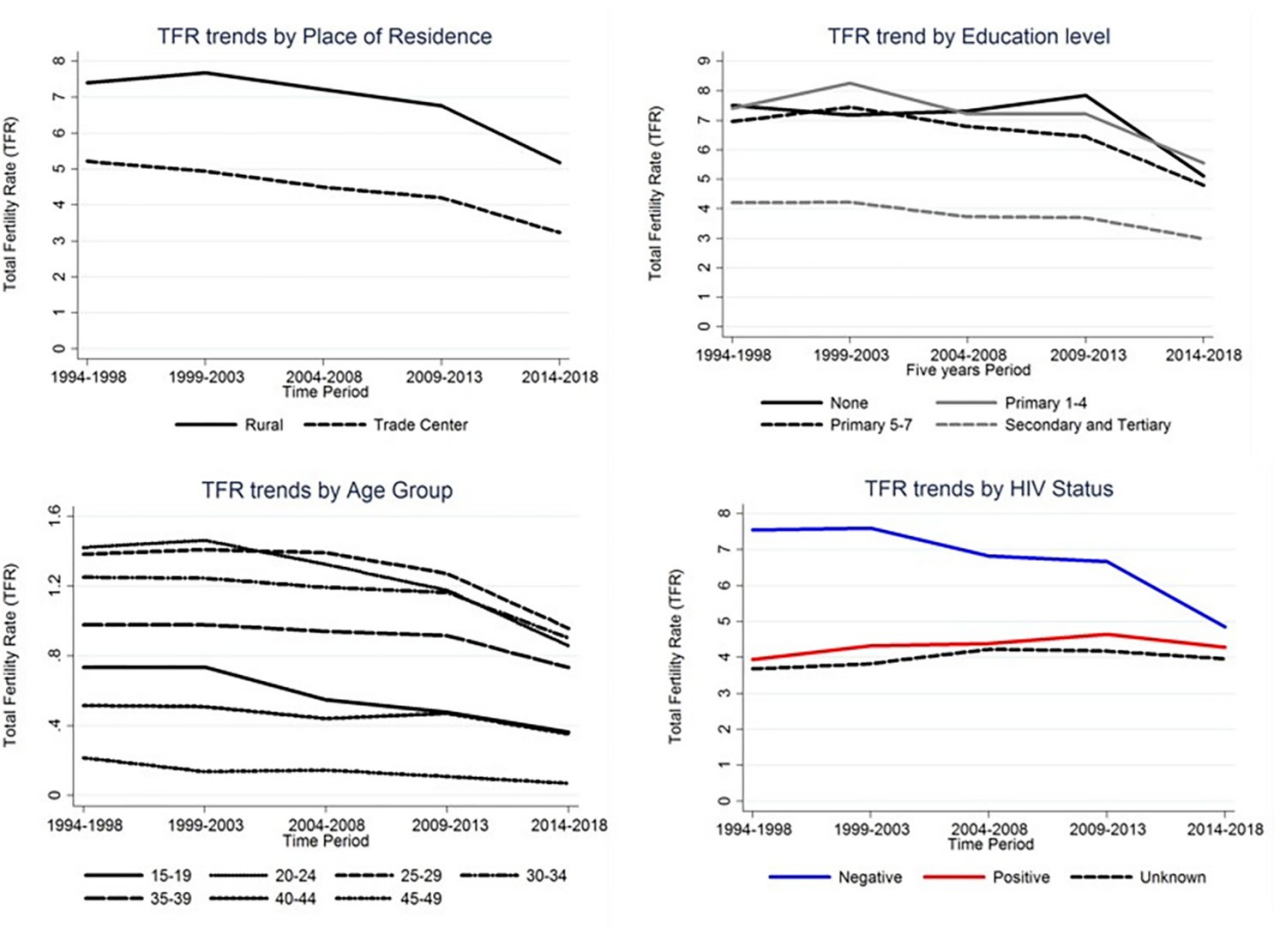
TFR by demographic characteristics. TFR by Place of residence (1994–2018). TFR by Place of Education (1994–2018). TFR by Age Groups (1994–2018). TFR by HIV status (1994–2018).

The overall TFR for women living with HIV was 4.4 births per woman compared to 6.7 births per woman in HIV-uninfected women, which is equivalent to a 42% difference in TFR (HR = 0.581; 95% CI: 0.574–0.624; p<0.001). The five-year TFR trends by HIV status showed a steeper decline in the TFR of HIV-uninfected women than women living with HIV. The steepest decline among HIV uninfected women was from 1994–1998 (7.6 births per woman) to 2014–2018 (4.9 births per woman). TFR was relatively unchanged over time in HIV infected women. TFR changed from 3.9 births per woman in 1994–1999 to 4.3 births per woman in 1999–2003, 4.4 births per woman in 2004–2008, to 4.6 births per woman in 2009–2013, and then to 4.3 births per woman in 2014–2018. TFR trends by education level, place of residence, age group, and HIV status are also summarized (Fig 1).

**Fig 2:**
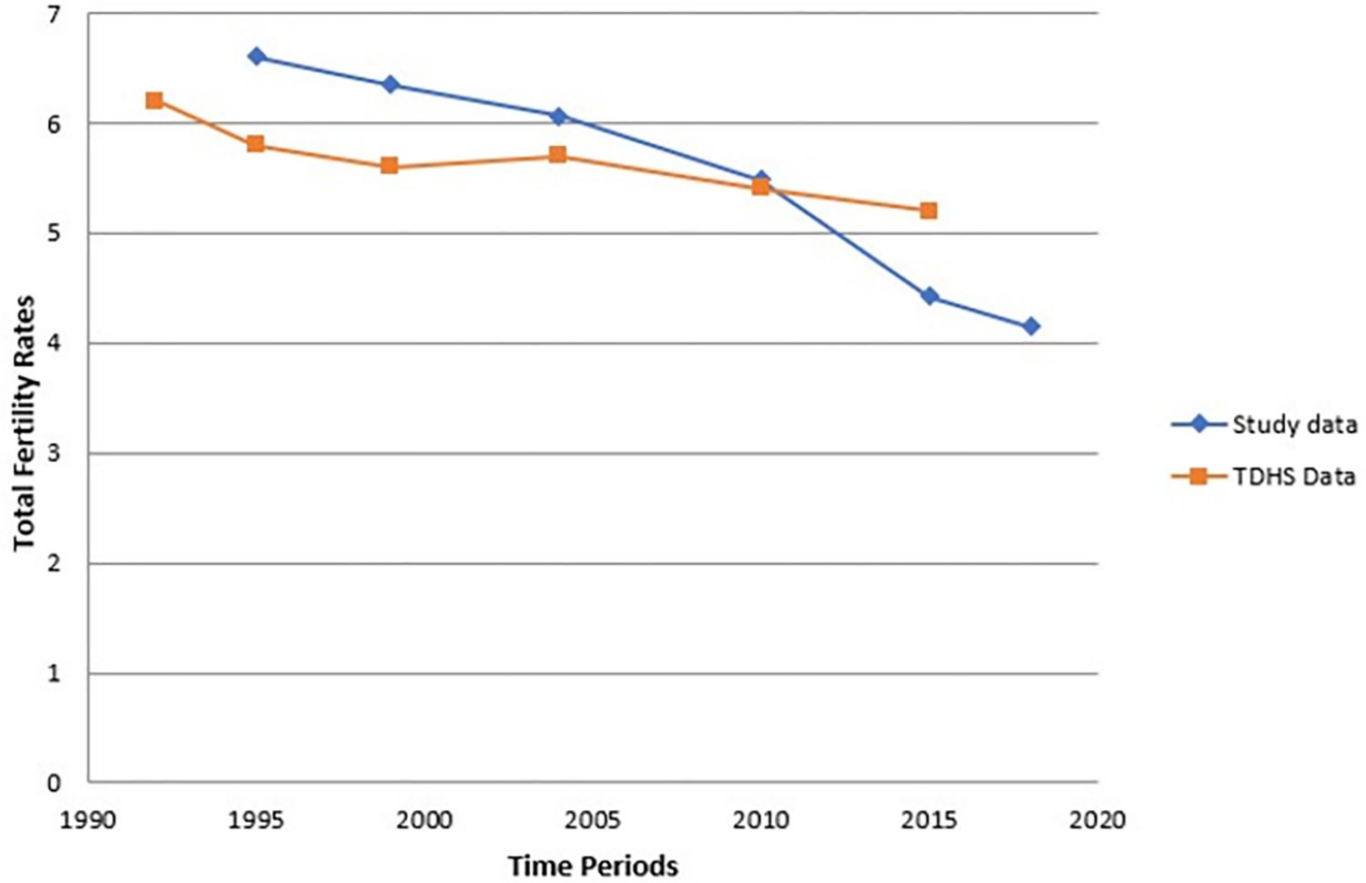
Comparing study TFR estimates against TDHS data.

We compared our results with fertility trends obtained in the previous National Tanzania Demography and Health Surveys (TDHS). TDHS showed a decline in fertility from 6.2 births per woman in 1992 to 5.2 births per woman. The decline in TFR shown in this paper is steep in the last 5-year period, and the comparison of TFR estimates against TDHS is shown (Fig 2).

### Age-specific fertility rates (ASFR)

**Fig 3:**
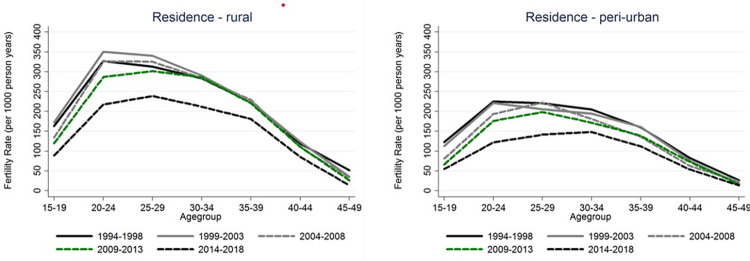
ASFR trends by place of place residence. ASFR by Calendar years (Rural), ASFR by Calendar years (Urban).

In general, patterns of ASFRs were different across different calendar years. The patterns changed slightly over time; with the peak ASFRs shifting towards older ages and decreasing as time progressed. This has been illustrated in rural and peri-urban residences plots (Fig 3).

**Fig 4:**
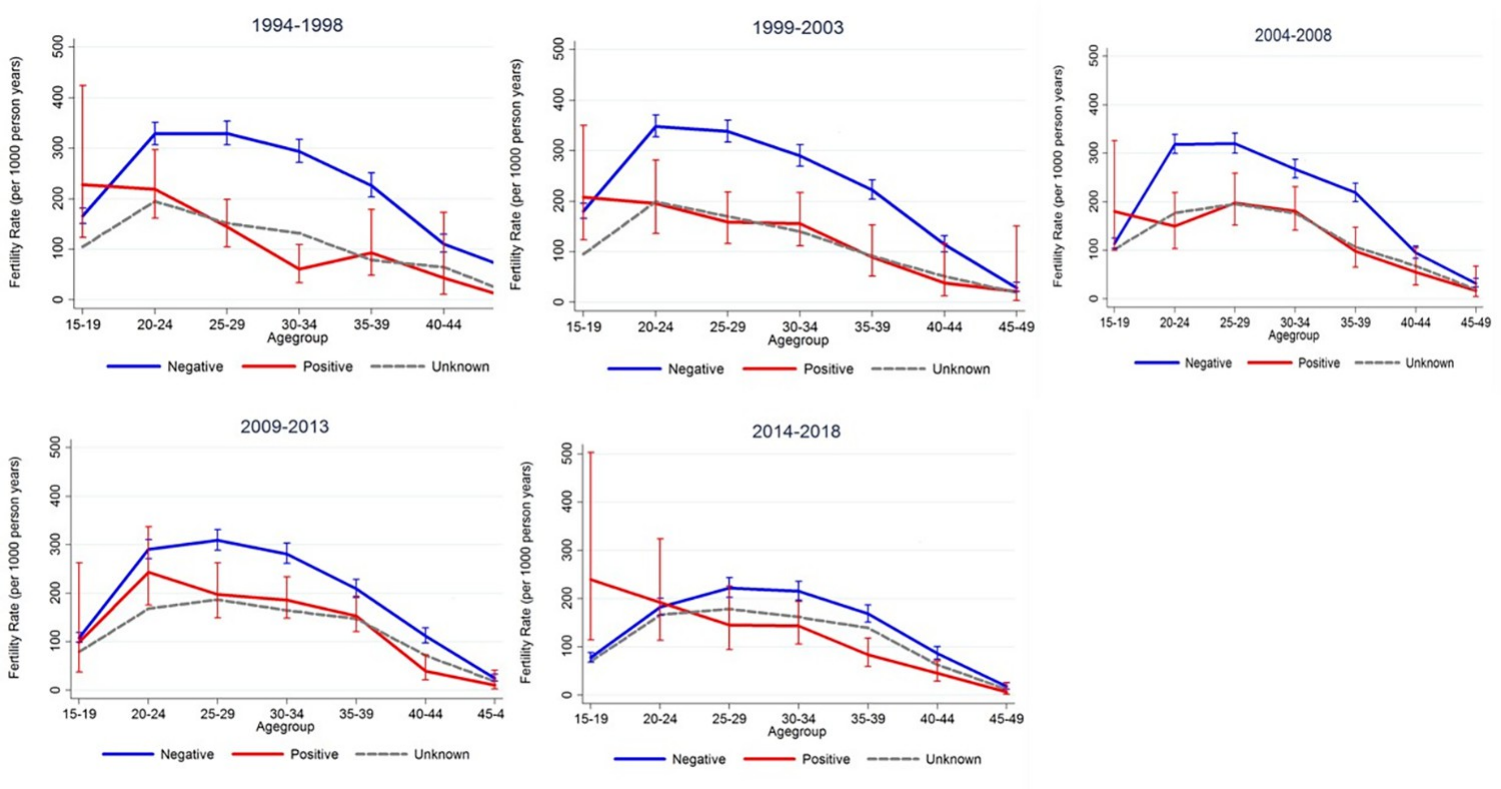
ASFR trends by HIV status. ASFR by HIV Status (1994–1998), ASFR by HIV Status (1999–2003), ASFR by HIV Status (2004–2008), ASFR by HIV Status (2009–2013), ASFR by HIV Status (2014–2018).

ASFR by HIV status also showed similar patterns (Fig 4). ASFR patterns are generally higher in HIV negative women compared to HIV positive with exception in adolescent women. ASFR are decreasing as time progressed; with the peak ASFRs shifting towards older ages and this can be visualized in 2014–2018 time periods.

### Impact of HIV on fertility

Various factors were found to be associated with changes in fertility among women living with HIV: peri-urban place of residence (age-adjusted HR = 0.727; 95% CI0.632–0.837; p<0.0001) and higher educational attainment (age-adjusted HR = 0.704; 95% CI0.498–0.995; p = 0.047). The fertility rate in peri-urban areas was 27% lower than women residing in rural areas and 30% lower in women who attended higher levels of education compared to those who did not attend any school. Therefore, fertility is generally low in women residing in urban settlements and those who attained higher education levels (Table 2).

**Table 2:** Factors associated with fertility changes among women aged 15–49 between January 1994to December 2018 by HIV status.

		HIV-Negative			HIV-Positive			Unknown HIV-status		
Variable	Category	Age-adjusted fertility hazard ratio^3^	95% Confidence Intervals (95% CI)	LRT p- value^2^	Age-adjusted fertility hazard ratio^3^	95% Confidence Intervals (95% CI)	LRT p value^2^	Age-adjusted fertility hazard ratio^3^	95% Confidence Intervals (95% CI)	LRT p value^2^
**Calendar year** ^**1**^										
	1994–1998	Ref			Ref			Ref		
	1999–2003	1.021	0974–1.070	0.431	1.153	0.896–1.485	0.269	1.027	0.924–1.141	0.624
	2004–2008	0.907	0.865–0.950	<0.001	1.236	0.975–1.565	0.080	1.053	0.951–1.166	0.319
	2009–2013	0.874	0.834–0.917	<0.001	1.443	1.147–1.816	0.002	1.006	0.914–1.107	0.904
	2014–2018	0.633	0.599–0.669	<0.001	1.092	0.842–1.418	0.504	0.954	0.873–1.042	0.296
**Place of Residence**										
	Rural	Ref			Ref			Ref		
	Peri urban	0.717	0.693–0.741	<0.001	0.727	0.632–0.837	<0.001	0.586	0.560–0.614	<0.001
**Educational level**										
	None	Ref			Ref			Ref		
	Primary 1–4	1.044	0.989–1.101	0.120	0.836	0.638–1.096	0.195	1.109	0.896–1.372	0.341
	Primary 5–7	0.924	0.889–0.960	<0.001	0.960	0.811–1.135	0.631	0.947	0.838–1.069	0.379
	Secondary and Tertiary	0.425	0.394–0.457	<0.001	0.704	0.498–0.995	0.047	0.403	0.305–0.532	<0.001
**ART periods**										
	>5 years Pre ART	Ref			Ref			Ref		
	5–0 years Pre ART	0.950	0.910–0.992	0.021	1.127	0.890–1.428	0.319	1.066	0.967–1.174	0.197
	ART	0.892	0.857–0.928	<0.001	1.427	1.167–1.744	0.001	1.043	0.957–1.137	0.331
	ART & Option B+	0.641	0.613–0.673	<0.001	1.099	0.870–1.387	0.430	0.956	0.881–1.036	0.273

^1^Time period is grouped into five years’ groups.

^2^Likelihood ratio test p-value

^3^Fertility hazard ratios are derived from the Cox regression model and are adjusted for age only.

When calendar years were disaggregated into periods of ART availability and analysis was confined to women living with HIV only, fertility during the period of ART introduction (2004–2008) was higher than Pre ART periods (age-adjusted HR = 1.43;95% CI 1.167–1.744, P<0.001). Fertility in other ART time periods (2013–2018) was not significantly different from fertility during the Pre–ART period (1994–1998) (age-adjusted HR = 1.09; 95% CI 0.870–1.387 p = 0.430). Details on the factors associated with fertility changes by HIV status are summarized (Table 2).

The final multivariable cox proportional hazard models (cox regression) included the effect of place of residence, time periods, HIV status, educational level, ART periods with and without the interactions between time periods and HIV status. The final multivariable model was obtained by testing evidence of interactions using likelihood ration test (Wald test) between two models. Wald test revealed no evidence of interactions, p> 0.05.

Therefore, the final multivariable analysis using the Cox proportional hazard model, with both HIV-positive and HIV-negative women combined, peri urban residence (adjusted HR = 0.768; 95% CI 0.743–0.794); p<0.0001), higher education attainment (adjusted HR = 0.560;95% CI 0.520–0.605); p<0.0001) and HIV infection (adjusted HR = 0.645; 95% CI 0.600–0.694; p<0.0001) were found to be independent risk factors for fertility changes. This is equivalent to 36% lower fertility amongst women living with HIV compared to HIV uninfected women, 24% lower fertility amongst women in peri-urban areas compared to women residing in rural areas and 46% lower fertility amongst women who attended high education compared to women who hadn’t.

Fertility rates (HIV status combined) were lower during the period of ART and PMTCT- Option B+ availability (2013–2018) compared to pre-ART periods (1994–1998) (adjusted HR = 0.804; 95% CI 0.707–0.922); p = 0.002). Details on the factors associated with fertility changes when HIV statuses are combined are summarized (Table 3).

**Table 3:** Independent factors associated with fertility changes among women aged 15–49 years (1994–2018): Multivariable analysis.

		Age-adjusted ^2^			Adjusted for all variables in the model^3^		
Variable	Category	hazard ratio	95% Confidence Intervals (95% CI)	LRT p-value	hazard ratio	95% Confidence Intervals (95% CI)	LRT p- value
**Calendar year** ^**1**^							
	1994–1998	Ref			Ref		
	1999–2003	1.009	0.968–1.052	0.676	1.125	1.041–1.215	0.003
	2004–2008	0.917	0.880–0.956	<0.001	1.036	0.932–1.151	0.512
	2009–2013	0.847	0.812–0.883	<0.001	1.102	0.910–1.149	0.705
	2014–2018	0.642	0.616–0.669	<0.001	0.936	0.809–1.084	0.378
**Place of Residence**							
	Rural	Ref			Ref		
	Peri urban	0.628	0.611–0.644	<0.001	0.768	0.743–0.794	<0.001
**Educational level**							
	None	Ref			Ref		
	Primary 1–4	1.086	1.033–1.143	0.001	1.040	0.986–1.096	0.151
	Primary 5–7	0.961	0.928–0.996	0.027	0.975	0.939–1.012	0.183
	Secondary and Tertiary	0.442	0.412–0.473	<0.001	0.560	0.520–0.605	<0.001
**HIV status**							
	HIV-Negative	Ref			Ref		
	HIV-Positive	0.582	0.542–0.624	<0.001	0.645	0.600–0.694	<0.001
	Unknown HIV Status	0.607	0.591–0.624	<0.001	0.798	0.741–0.859	<0.001
**ART periods** ^**4**^							
	>5 years Pre-ART	Ref			Ref		
	5–0 years Pre-ART	0.958	0.958–0.996	0.033	0.919	0.851–0.993	0.033
	ART	0.885	0.854–0.916	<0.001	1.010	0.908–1.122	0.855
** **	ART & Option B+	0.642	0.619–0.669	<0.001	0.804	0.702–0.922	0.002

^1^Time period is grouped into five years’ groups.

^2^Age adjusted hazard ratios are derived from the Cox regression model

^3^Adjusted hazard ratios are derived from the Cox regression model. They are adjusted for Age, Place of Residence, Education level, Marital status, and ART Period.

^4^ART periods;>5 years Pre-ART (1994–2000: 5–0 years Pre-ART (2006–2012): ART & Option B+ (2013–2018)

## Discussion

In our study using Health and Demographic Surveillance data, we found a decline in TFR from 1994 to 2018 among all women aged 15–49 years, with a steeper decline in more recent years (2009–2013 to 2014–2018). Overall, it remains clear that women living with HIV have significantly lower fertility rates than HIV-negative women. However, the fertility rates in women living with HIV infection remained similar across the time periods. Introduction of ART coincided with a higher fertility rate in women living with HIV, but this was not significant and there was no a fertility restoration among women living in our study area.

We found various other factors that also influenced the reductions in fertility over this time period, including residence in urban areas and attainment of high levels of education. Our findings support others in recognizing the relationship between an increase in a women’s education level and its associated reduction in fertility amongst younger women [28–31]. In Tanzania, access to secondary education has been increasing from 2010 onwards, and in 2015, the government implemented universal access to secondary education [32]. The steeper change is also likely to be a result of the increased access to and uptake of family planning in marital but primarily extramarital unions and the increased levels of urbanization within the study site [33]. Our findings support others in Tanzania [2, 3] and from other countries within SSA [34], who found that women living in rural areas had higher fertility rates than those living in urban areas and lower fertility in highly educated women than uneducated women.

Compared to HIV-uninfected women, the fertility rates in women living with HIV were consistently lower. A Malawian study conducted in 2017 showed a fertility reduction of 8% in women living with HIV compared to HIV-uninfected women [35], with similar results reported in 2014 by a large study involving eight countries in West Africa [36] and of another involving three countries in East and Southern Africa [13]. However, we found that the trend in decline in fertility rates differed by HIV status and was steeper in HIV-uninfected women than in women living with HIV. In 2017, Marston and colleagues demonstrated similar results across 46 household surveys in the SSA. The findings suggested that high ART coverage attenuated the relationship between HIV and fertility in women living with HIV [13, 18]. ART restores the lost fertility potential under background trends of declining fertility amongst the general population of women in Tanzania. The population-level impact of ART on fertility depends on the coverage of ART (primarily through the PMTCT-Option B+ program) and the percentage of people knowing their HIV status (measured by HIV testing rates).

Studies in other East African countries (Uganda and Kenya) found no significant relationship between ART use and incident pregnancy (cause-specific hazard ratio: 0.98; 95% CI: 0.91 to 1.05), which is consistent with the results we show. Women at enrolment and on ART had an increased risk of incident pregnancy compared to those at enrolment and not on ART (cause-specific hazard ratio: 1.11; 95% CI: 1.01 to 1.23) [19]. Another study in Tanzania using national DHS data reported an increase in fertility among ART users [37]. Child-bearing behaviour is influenced by women’s fertility intentions or desire to bear more children, which in turn is influenced by social-cultural norms and traditions. On-going research in the Magu HDSS is further exploring trends in fertility desires over time to investigate how fertility desire is changing in the context of the ART roll out.

The national Tanzania Demography and Health Surveys showed a decline in fertility from 6.2 births per woman in 1992 to 5.2 births per woman in 2015 [2].The decline in TFR shown in this paper is steep in the last 5-year period. Another study in the Magu HDSS showed a contraceptive rate of 30% [28], which would indicate an expected TFR of approximately 5 children per woman. This paper reports a lower TFR than expected, which may be due to some births being missed in the DSS. We have explored this data quality issue and cannot find any potential reason for this, but later rounds of the DSS may help to uncover any systematic problems in births records. The lower-than-expected fertility of 4.3 births per woman in the 2014–2018 periods might be plausible. The HDSS has experienced substantial urban immigration, more people are living in Kisesa but are working in the Mwanza city. This might have increased the proportion of peri-urban residents and those with higher education women in the HDSS population. Second, the majority of the births used to occur in young women aged 15–24 years, which showed the steepest decline in fertility levels. Over the 25 years of the study data, the peak in fertility was seen in older age groups.

### Strengths and limitations of the study

This study’s strengths lie in the large number of women and a long period of follow-upwith 145 452 PY of follow-up from 1994 to 2018, which permitted meaningful epidemiological comparisons. Additionally, the study had objective measures of outcome and exposure variables, with births collected from household visits and HIV status being measured in the field using the standardized laboratory testing protocol in Tanzania. However, various weaknesses should be noted when interpreting the study findings. First, the changing characteristics of the HDSS population included overrepresentation of female study participants over males in the epidemiological serological surveillance. Second, the HDSS population may not represent the population of Tanzania as the Country. HDSS fertility trends over the 1994–2016 periods are similar to the TDHS fertility estimates. Third, there may be other potential confounders that could have influenced the association between HIV infection and fertility, although the confounding variables in this dataset were controlled for during analysis. We assumed the date of HIV seroconversion at the mid-point between the last HIV negative test and the first HIV positive test, and in the absence of an HIV positive test, we assumed that those women remained HIV negative for five years after the last HIV negative test. We did not undertake a sensitivity analysis of these assumptions.

## Conclusion

We reported a fertility decline among women in the study area from 1994 to 2018. The downward trends are most likely caused by increased female education and urbanization. These downward fertility trends will continue due to increasing urbanization, female education. The difference in fertility by HIV status will continue to narrow as population fertility continues to decline in Tanzania. We suspect constancy of fertility among women living with HIV was due to use of female contraception compared to HIV uninfected women.

These updated results on fertility trends by HIV status could improve the estimations of population HIV prevalence from antenatal HIV data sources. The fertility rate among women living with HIV will support the need to integrate and promote family planning services within the PMTCT program. From the policy perspective, the findings suggest that policies to increase female education attainment and promote access to and uptake of female contraceptives will accelerate the decline in fertility.

## References

[1] United Nation, Department of Economic and Social Affairs. Population Dision (2020), World Fertility 2019. Early and late childbearing among adolescent women (ST/ESA/SER.A/446).

[2] Ministry of Health, Community Development, Gender, Elderly and Children (MoHCDGEC) [Tanzania Mainland], Ministry of Health (MoH) [Zanzibar], National Bureau of Statistics (NBS), Office of the Chief Government Statistician (OCGS) and I. Tanzania Demographic and Health Survey and Malaria Indicator Survey (TDHS-MIS) 2015–16. 2016.

[3] Hunter SC, Isingo R, Boerma JT, Urassa M, Mwaluko GM, Zaba B. The association between HIV and fertility in a cohort study in rural Tanzania. J Biosoc Sci. 2003 Apr;35(2):189–99. doi: 10.1017/s0021932003001895 12664957

[4] Saleem H. T., Surkan P. J., Kerrigan D., & Kennedy C. E. (2019). “If I don’t have children, they will know that I’m sick”: fertility desires of women and men living with HIV in Iringa, Tanzania. *AIDS Care—Psychological and Socio-Medical Aspects* of AIDS/HIV, 31(7), 908–911. 10.1080/09540121.2019.1576844 30712359

[5] UNAIDS. Data 2017. Program HIV/AIDS. 2017;1–248.

[6] United Republic of Tanzania. December 2017 Tanzania Hiv Impact Survey. 2017.

[7] Dublin S, Blatner WA, White GC 2nd, Goedert JJ. Procreation and HIV. Vol. 342, Lancet (London, England). England; 1993. p. 1241–2. 10.1016/0140-6736(93)92225-i 7901561

[8] Fleming DT, Wasserheit JN. From epidemiological synergy to public health policy and practice: the contribution of other sexually transmitted diseases to sexual transmission of HIV infection. Sex Transm Infect. 1999 Feb;75(1):3–17. doi: 10.1136/sti.75.1.3 ; PMCID: PMC1758168. 10448335 PMC1758168

[9] Nakiganda LJ, Agardh A, Asamoah BO. Cross-sectional study on the prevalence and predictors of pregnancy among women living in HIV discordant relationships in a rural Rakai cohort, Uganda. BMJ Open. 2018 Apr 24;8(4):e019818. doi: 10.1136/bmjopen-2017-019818 Erratum in: BMJ Open. 2018 Jun 30;8(6): ; PMCID: PMC5922486. 29691244 PMC5922486

[10] Lewis JJ, Ronsmans C, Ezeh A, Gregson S. The population impact of HIV on fertility in sub-Saharan Africa. AIDS. 2004 Jun;18 Suppl 2:S35–43. doi: 10.1097/00002030-200406002-00005 . 15319742

[11] Ross A, Van der Paal L, Lubega R, Mayanja BN, Shafer LA, Whitworth J. HIV-1 disease progression and fertility: the incidence of recognized pregnancy and pregnancy outcome in Uganda. AIDS. 2004 Mar 26;18(5):799–804. doi: 10.1097/00002030-200403260-00012 . 15075516

[12] Marston M, Zaba B, Eaton JW. The relationship between HIV and fertility in the era of antiretroviral therapy in sub-Saharan Africa: evidence from 49 Demographic and Health Surveys. Trop Med Int Health. 2017 Dec;22(12):1542–1550. doi: 10.1111/tmi.12983 Epub 2017 Oct 24. ; PMCID: PMC5716842. 28986949 PMC5716842

[13] Marston l; Nakiyingi-Miiro Jessica; Kusemererwa Sylvia; Urassa Mark; Michael Denna; Nyamukapa Constance; et al. The effects of HIV on fertility by infection duration: evidence from African population cohorts before antiretroviral treatment availability. AIDS 31():p S69–S76, April 2017. | doi: 10.1097/QAD.0000000000001305 28296802

[14] Fabiani M, Nattabi B, Ayella EO, Ogwang M, Declich S. Differences in fertility by HIV serostatus and adjusted HIV prevalence data from an antenatal clinic in northern Uganda. Trop Med Int Health. 2006 Feb;11(2):182–7. doi: 10.1111/j.1365-3156.2005.01554.x . 16451342

[15] Gray RH, Li X, Kigozi G, Serwadda D, Brahmbhatt H, Wabwire-Mangen F, et al. Increased risk of incident HIV during pregnancy in Rakai, Uganda: a prospective study. Lancet. 2005 Oct 1;366(9492):1182–8. doi: 10.1016/S0140-6736(05)67481-8 . 16198767

[16] Desgrées du Loû A, Msellati P, Yao A, Noba V, Viho I, Ramon R, et al. Impaired fertility in HIV-1-infected pregnant women: a clinic-based survey in Abidjan, Côte d’Ivoire, 1997. AIDS. 1999 Mar 11;13(4):517–21. doi: 10.1097/00002030-199903110-00011 . 10197381

[17] Yeatman S, Eaton JW, Beckles Z, Benton L, Gregson S, Zaba B. Impact of ART on the fertility of HIV-positive women in sub-Saharan Africa. Trop Med Int Health. 2016 Sep;21(9):1071–85. doi: 10.1111/tmi.12747 Epub 2016 Jul 22. . 27371942

[18] Marston M, Nakiyingi-Miiro J, Hosegood V, Lutalo T, Mtenga B, Zaba B, et al. (2016) Measuring the Impact of Antiretroviral Therapy Roll-Out on Population Level Fertility in Three African Countries. PLoS ONE 11(3): e0151877. doi: 10.1371/journal.pone.0151877 27015522 PMC4807830

[19] Elul B, Wools-Kaloustian KK, Wu Y, et al. Untangling the Relationship Between Antiretroviral Therapy Use and Incident Pregnancy: A Marginal Structural Model Analysis Using Data From 47,313 HIV-Positive Women in East Africa. Journal of Acquired Immune Deficiency Syndromes (1999). 2016 Jul;72(3):324–332. doi: 10.1097/QAI.0000000000000963 ; PMCID: PMC4911268. 26910499 PMC4911268

[20] Kabami J., Turyakira E., Biraro S. et al. Increasing incidence of pregnancy among women receiving HIV care and treatment at a large urban facility in western Uganda. *Reprod Health* 11, 81 (2014). 10.1186/1742-4755-11-81. PMC4364564 25480367

[21] Lancaster KE, Kwok C, Rinaldi A, Byamugisha J, Magwali T, Nyamapfeni P, et al. Incident pregnancy and pregnancy outcomes among HIV-infected women in Uganda and Zimbabwe. Int J Gynaecol Obstet. 2015 Dec;131(3):255–9. doi: 10.1016/j.ijgo.2015.06.035 Epub 2015 Sep 8. ; PMCID: PMC4661121. 26387468 PMC4661121

[22] NACP. The global AIDS Response Country progress report,. Tanzania Ministry of Health. 2014.

[23] Ministry of Health, Community Development, Gender E and C (MoHCDGEC). National Guidelines for Management of HIV and AIDS. United Republic of Tanzania. 2015.

[24] Ministry of Health, Community Development, Gender, Elderly and Children (MoHCDGEC) [Tanzania Mainland], Ministry of Health (MoH) [Zanzibar], National Bureau of Statistics (NBS), Office of the Chief Government Statistician (OCGS) and I 2016. Tanzania Demographic and Health Survey and Malaria Indicator Survey 2015–2016 (TDHS-MIS).

[25] Kishamawe C, Isingo R, Mtenga B, Zaba B, Todd J, Clark B, et al. Health & Demographic Surveillance System Profile: The Magu Health and Demographic Surveillance System (Magu HDSS). Int J Epidemiol. 2015 Dec;44(6):1851–61. doi: 10.1093/ije/dyv188 Epub 2015 Sep 24. ; PMCID: PMC4911678. 26403815 PMC4911678

[26] Wambura M, Urassa M, Isingo R, Ndege M, Marston M, Slaymaker E, et al., HIV prevalence and incidence in rural Tanzania: results from 10 years of follow-up in an open-cohort study. J Acquir Immune Defic Syndr. 2007 Dec 15;46(5):616–23. doi: 10.1097/QAI.0b013e31815a571a ; PMCID: PMC2842883. 18043316 PMC2842883

[27] Boerma JT, Urassa M, Senkoro K, Klokke A, Ngẃeshemi JZ. Spread of HIV infection in a rural area of Tanzania. AIDS. 1999 Jul 9;13(10):1233–40. doi: 10.1097/00002030-199907090-00013 . 10416528

[28] Safari W., Urassa M., Mtenga B. et al. Contraceptive use and discontinuation among women in rural North-West Tanzania. Contracept Reprod Med 4, 18 (2019). doi: 10.1186/s40834-019-0100-6 31754451 PMC6852765

[29] Kebede E, Goujon A, Lutz W. Stalls in Africa’s fertility decline partly result from disruptions in female education. Proc Natl Acad Sci U S A. 2019;116(8):2891–6. doi: 10.1073/pnas.1717288116 30718411 PMC6386713

[30] Raftery AE, Liu DH. Accelerating fertility decline through education and family planning. N-IUSSPORG. 2021; 10.1111/padr.12347 PMC7590131 33132461

[31] Liu DH, Raftery AE. How Do Education and Family Planning Accelerate Fertility Decline? Popul Dev Rev. 2020;46(3):409–41. doi: 10.1111/padr.12347 33132461 PMC7590131

[32] Ministry of Education. Universal Secondary School Education in Tanzania. 2014.

[33] United Republic of Tanzania. 2012 Population and Housing Census. Vol. IV. 2015.

[34] Odimegwu CO, Akinyemi JO, Banjo OO, Olamijuwon E, Amoo EO. Fertility, Family Size Preference and Contraceptive Use in Sub-Saharan Africa: 1990–2014. Afr J Reprod Health. 2018 Dec;22(4):44–53. doi: 10.29063/ajrh2018/v22i4.5 . 30632721

[35] McLean E, Price A, Chihana M, Kayuni N, Marston M, Koole O, et al. Changes in Fertility at the Population Level in the Era of ART in Rural Malawi. J Acquir Immune Defic Syndr. 2017 Aug 1;75(4):391–398. doi: 10.1097/QAI.0000000000001395 ; PMCID: PMC5483985. 28653969 PMC5483985

[36] Burgos-Soto J, Balestre E, Minga A, Ajayi S, Sawadogo A, Zannou MD, et al. Incidence of pregnancy after antiretroviral therapy initiation and associated factors in 8 West African countries. J Acquir Immune Defic Syndr. 2014 Oct 1;67(2):e45–54. doi: 10.1097/QAI.0000000000000279 ; PMCID: PMC4166575. 25216079 PMC4166575

[37] Mbita G, Renju J, Lija G, Conserve DF, Todd J. Effect of antiretroviral therapy on fertility rate among women living with HIV in Tabora, Tanzania: An historical cohort study. PLoS One [Internet]. 2019;14(9):1–14. Available from: 10.1371/journal.pone.0222173 PMC6730877 31491017

